# Oxalate as an Emerging Contributor to Cardiovascular Disease: Links to Inflammation, Immunity, and Oxidative Stress

**DOI:** 10.3390/nu18081190

**Published:** 2026-04-10

**Authors:** Mary A. E. M. Doamekpor, Vivek Verma, Christine M. Wright, Breanna Young, Diksha S. Saini, Gregory A. Payne, Clintoria R. Williams, Tanecia Mitchell

**Affiliations:** 1 Department of Urology, University of Alabama at Birmingham, Birmingham, AL 35294, USA; mdoam5@uab.edu (M.A.E.M.D.); vverma@uabmc.edu (V.V.); cwright7@uab.edu (C.M.W.); breyoung@uab.edu (B.Y.); 2 Department of Cell Biology, Neuroscience and Physiology, Wright State University, Dayton, OH 45435, USA; saini.71@wright.edu (D.S.S.); clintoria.williams@wright.edu (C.R.W.); 3 Department of Medicine, University of Alabama at Birmingham, Birmingham, AL 35294, USA; gpayne@uabmc.edu

**Keywords:** oxalate, calcium oxalate, cardiovascular disease, metabolism, immunity, inflammation, atherosclerosis, reactive oxygen species

## Abstract

Cardiovascular disease (CVD) is the world’s leading cause of death and continues to rise in prevalence, contributing to healthcare and economic costs. Following diagnosis, patients are advised to adopt medication regimens, increase physical activity, and modify dietary intake to reduce disease progression and prevent additional comorbidities. Oxalate is a small molecule in plant-derived foods such as spinach, potatoes, almonds, and peanuts and is also produced endogenously. Although oxalate is traditionally studied in the context of kidney stone disease, recent evidence suggests that it may be a dietary contributor to inflammation and oxidative stress in CVD. Elevated systemic oxalate levels promote reactive oxygen species (ROS) generation and activate inflammatory pathways such as nuclear factor-kappa B (NF-κB), mitogen-activated protein kinase (MAPK), and the NLRP3 inflammasome, which are key players in CVD. In this narrative review, we discuss the current literature describing the role of inflammation in CVD and evaluate emerging evidence that dietary oxalate may influence immune, oxidative, and vascular mechanisms contributing to CVD development and progression. In addition, we highlight populations that may be most vulnerable to oxalate-mediated vascular effects. We conclude by describing existing gaps in knowledge and potential future directions for the field. Understanding these mechanisms further may guide dietary recommendations and delineate oxalate’s potential role as a modifiable risk factor for CVD.

## 1. Introduction

Cardiovascular disease (CVD) encompasses a group of conditions, including atherosclerosis, coronary artery disease (CAD), and cerebrovascular disease, that negatively affect the heart and blood vessels. CVD is becoming more prevalent worldwide and significantly impacts both healthcare and economic costs. Further, CVD accounts for over 17 million deaths annually [1] with atherosclerosis being the leading underlying cause of death [2]. The stages of atherosclerosis include vascular endothelial dysfunction [3], fatty streak formation, fibrous plaque formation, and plaque rupture [4]. In its advanced stages, atherosclerosis can lead to hypertension, stroke, myocardial infarction (MI), and sudden cardiac death (SCD) [5]. Importantly, oxidative stress and chronic inflammation are key drivers across these disease processes [6,7,8,9,10].

Although inflammation elicits protective mechanisms to remove cellular debris and initiates tissue repair, dysregulated immune responses can disrupt vascular homeostasis and further enhance inflammatory signaling [11,12]. Several endogenous and exogenous factors such as acute/chronic infection, mental stress, smoking, and diet are all reported to stimulate inflammation during CVD development and progression [5,13]. In addition, it is well established that consuming highly processed foods or meals rich in saturated fats [14,15], sodium [16], or oxalate [17,18] are all associated with an increased risk of CVD.

Oxalate is found in plant-derived foods such as spinach, potatoes, almonds, and peanuts in significant amounts. It is also the primary component of the most common type of kidney stones. Increased urinary and systemic oxalate levels have been implicated in chronic kidney disease (CKD) progression [19,20]. Further, both kidney stone disease and CKD are independently associated with increased CVD risk [21,22,23]. The underlying mechanisms of these associations are not well defined but are thought to involve inflammation, oxidative stress, disruptions in mineral metabolism, and endothelial dysfunction.

Growing interest in oxalate biology is due to increased consumption of certain plant-based foods that are oxalate rich [19,20], increased kidney stone prevalence [21], and accumulating evidence demonstrating that oxalate activates reactive oxygen species (ROS) [22,23] and inflammatory signaling pathways [24] central to CVD. These mechanistic links suggest that high dietary oxalate intake could influence immune pathways related to CVD. Recent studies have shown associations between oxalate, vascular injury [25], and CVD events [17,26,27], supporting the importance of evaluating dietary oxalate within a CVD context.

Here, we describe the role of inflammation in select cardiovascular diseases (CVDs) and examine how oxalate-rich meals could influence these processes. A deeper understanding of these mechanisms is needed, as high dietary oxalate intake may contribute to the development or progression of CVD. Despite growing awareness of diet-related inflammation in CVD, dietary compounds that independently activate immune and oxidative pathways outside of traditional macronutrients remain understudied.

## 2. Pathogenesis of Atherosclerosis and Immunity

**Figure 1:**
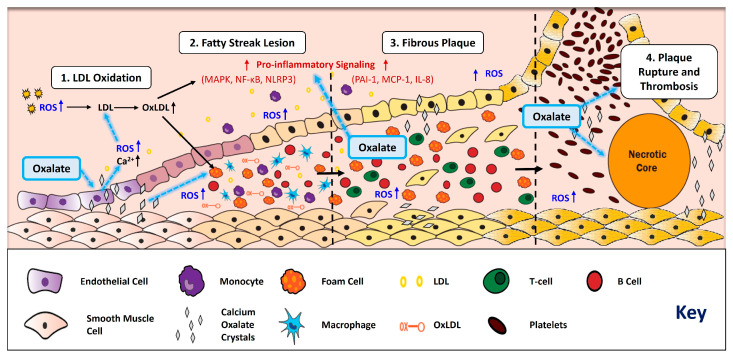
Proposed intersections between oxalate-associated mechanisms and key stages of atherosclerosis. The main steps of atherosclerosis include LDL oxidation, fatty streak lesion formation, fibrous plaque growth, and plaque rupture with thrombosis. Superimposed on this framework, oxalate (both soluble (NaOx) and insoluble (CaOx crystals)—blue boxes and silver diamonds, respectively) is proposed to amplify ROS production, calcium (Ca^2+^) release from endothelial cells, pro-inflammatory signaling (MAPK, NF-κB, NLRP3), immune cell recruitment, and plaque instability. Solid black arrows denote established mechanisms; light blue dashed arrows denote proposed intersecting mechanisms. ROS (shown in dark blue) highlight oxidative stress as a common mechanism. Pro-inflammatory pathways are shown in red.

Atherosclerosis is initiated by vascular endothelial dysfunction, characterized by loss of vascular homeostasis, impaired nitric oxide bioavailability, reduced vasodilation, and heightened inflammatory signaling [28]. This dysfunction causes low-density lipoprotein (LDL) particles to infiltrate the arterial intima (the innermost lining of the artery) [3,4]. Subsequently, LDL particles undergo chemical modification to oxidized LDL (oxLDL) through enzymatic and non-enzymatic processes driven by ROS [29,30] (Figure 1).

Internalization of oxLDL by vascular and immune cells amplifies ROS generation, promotes inflammatory signaling, and drives immune cell recruitment, leading to fatty streak formation, which marks the early development of atherosclerotic plaques (Figure 1). Over time, these fatty streak lesions evolve into fibrous plaques [31] that comprise a necrotic core containing lipids and dead cells (Figure 1) [4,32,33]. However, pro-thrombotic and inflammatory factors (plasminogen activator inhibitor-1 (PAI-1), monocyte chemoattractant protein-1 (MCP-1), and Interleukin-8 (IL-8)) can weaken the fibrous plaque and cause plaque rupture [34,35]. Subsequently, clotting factors and platelets aggregate around the plaque and can initiate coagulation and thrombus formation [36,37] (Figure 1), which may result in the development of advanced CVDs, such as MI and stroke.

The immune system plays a vital role in both promoting and mitigating atherosclerosis. Protective cells include regulatory T cells and M2 macrophages, whereas M1 macrophages and neutrophils exacerbate plaque formation and instability [38]. In addition, damage-associated molecular patterns (DAMPS) released from dying cells, are known to activate innate immunity and contribute to disease progression. Notably, DAMPS such as oxLDL particles, can increase the expression of adhesion molecules in the vascular endothelium, as well as promote the release of chemokines including CC-motif chemokine 2 and CXC-motif chemokine 1 [39,40,41,42,43]. These chemokines can recruit and differentiate monocytes from the bloodstream into M1 pro-inflammatory macrophages, which phagocytose oxLDL from the artery wall and transform into foam cells [44]. Vascular smooth muscle cells can also engulf oxLDL particles and become foam cells [45,46,47,48].

Inflammatory cytokines and chemokines also recruit neutrophils and dendritic cells to the endothelium. Neutrophils further accelerate atherosclerosis by releasing ROS and myeloperoxidases that oxidize LDL and exacerbate inflammation [49,50]. They also produce anti-microbial peptides such as cathelicidin and α-defensin to further stimulate macrophage activity [51,52,53]. However, a key aspect of neutrophils in atherosclerosis is their ability to form Neutrophil Extracellular Traps (NETs), which are large extracellular networks composed primarily of neutrophil DNA that capture and kill invading pathogens. NETs can also form in blood vessels and are implicated in thrombosis and vascular inflammation [54]. Cholesterol crystals and oxLDL can induce NET formation in neutrophil cells in vitro [55,56], which can facilitate LDL oxidation, aggregation, foam cell formation, and plaque instability [57]. It has been reported that mice prone to atherosclerosis (ApoE-deficient) fed a high fat diet, form NETs exclusively in cholesterol-rich regions of the aorta [55]. NETs also contain large amounts of histone H4, which can lyse vascular cells and promote plaque instability [58,59].

Dendritic cells bridge innate and adaptive immunity, which is primarily composed of T cells and B cells. Specifically, dendritic cells can phagocytose pathogens and foreign molecules, and upon maturation, present the antigens to T cells to initiate the adaptive immune response [60,61]. In fact, oxLDL can induce dendritic cell maturation [62] and cytokine secretion [63,64]. Activated T cells can then differentiate into various subsets, including pro-inflammatory Th1 and Th17 cells [65]. Th1 and Th17 cells secrete cytokines like interferon-gamma (IFN-γ), tumor necrosis factor-alpha (TNF-α), and Interleukin-17 (IL-17) that exacerbate inflammation [66,67]. Strikingly, Th17 secretion of IL-17 also stabilizes plaques and may prevent disease progression [68,69]. B cells can remove circulating lipids through the B cell receptor and low-density lipoprotein receptor and produce antibodies against oxLDL, further promoting inflammation and atherosclerosis [70,71]. Notably, many of these oxidative and inflammatory pathways central to atherosclerosis are also activated by oxalate exposure, suggesting mechanistic overlap between oxalate-induced immune activation and CVD.

## 3. Inflammatory Pathways and CVD

Multiple inflammatory pathways are activated in CVD, including the mitogen-activated protein kinase (MAPK) pathway, nuclear factor-kappa B (NF-κB) pathway, and inflammasomes, and all contribute collectively to disease development and progression (Figure 1). The MAPK pathway responds to diverse stimuli including cellular stress and cytokines that alter gene expression, metabolism, and apoptosis. In CVD, MAPK influences macrophage polarization, plaque stability, and vascular smooth muscle proliferation [72]. OxLDL also activates MAPK signaling and enhances macrophage oxLDL uptake during foam cell formation [73,74,75]. Importantly, consistent MAPK activation stimulates an inflammatory and oxidative environment that further promotes atherogenesis.

The NF-κB transcription factor is another modulator of inflammation activated by oxLDL, oxidative stress, and inflammatory cytokines [76,77]. Once activated, NF-κB induces the mRNA levels of pro-inflammatory genes, encoding cytokines, chemokines, and adhesion molecules [78]. Mice lacking IkappaB kinase 2 (an upstream activator of NF-κB) were shown to have increased atherosclerosis and reduced anti-inflammatory IL-10 cytokine levels, suggesting that NF-κB can also participate in inflammation resolution depending on the context [79]. NF-κB activation also upregulates NLR family pyrin domain containing 3 (NLRP3) protein, which recognizes and binds to DAMP and pathogen associated molecular pattern molecules, and assembles into an NLRP3 inflammasome, which produces pro-inflammatory cytokines, Interleukin-1β (IL-1β) and Interleukin-18 (IL-18) [80,81]. Cholesterol crystals, oxLDL, and DAMPS such as high-mobility group box 1 (HMGB1), heat shock protein 70 (HSP70), and S100 calcium-binding protein A12 (S100A12) are all potent activators of NLRP3 inflammasomes [82,83]. Additionally, macrophage uptake of oxLDL can release HSP70, and further stimulate cytokine secretion and inflammation [84]. Collectively, these pathways recruit immune cells to plaques and contribute to fibrous-cap thinning and plaque instability. These mechanisms underscore a distinct interplay between lipid metabolism, immune activation, and inflammatory signaling in the development of atherosclerosis and CVD.

## 4. The Role of Diet in Modulating Inflammation in CVD

Diet is a major determinant of cardiovascular health, shaping systemic inflammation, lipid metabolism, and vascular function. Here, we summarize how pro- and anti-inflammatory dietary patterns modulate these pathways and alter CVD risk.

### 4.1. Pro-Inflammatory Diets

Diets high in saturated and trans fatty acids are associated with higher LDL cholesterol, increased inflammation [85], and atherosclerosis [14]. Cholesterol is essential for the synthesis of vitamin D and steroid and sex hormones, as well as cell membrane structure [86]. However, excess cholesterol deposits in the vasculature can be detrimental to cardiovascular health [87]. In blood, cholesterol is carried by lipoproteins with a lipid-rich core (containing triglycerides and cholesterol esters) and hydrophilic outer membrane (containing apolipoprotein, phospholipids, and free cholesterol) [88]. The primary types of lipoproteins in the circulation include chylomicrons, very-low-density lipoproteins, intermediate-density lipoproteins, high-density lipoprotein, and LDL [86]. Among these, LDL is most strongly linked to CVD risk and serves as a primary clinical biomarker. Although diet is a major factor in modulating LDL cholesterol levels, genetic and lifestyle factors also play a crucial role [89].

Clinical evidence suggests that pro-inflammatory diets are associated with higher CVD risk. In a 32-year longitudinal dietary study of United States health professionals, the empirical dietary inflammatory pattern (EDIP), which integrates consumption of foods known to promote or attenuate inflammation, was used to evaluate correlations with diet and inflammation [13]. The EDIP score was determined by each participant’s self-reported intake of 18 specific diets. Diets high in processed meats and refined grains were positively associated with systemic inflammation, whereas higher intake of green leafy vegetables and whole grains was inversely associated. Individuals with the highest EDIP score had a 38% increased risk of CVD and significantly elevated inflammatory markers [13].

Similarly, another study analyzed 1303 adults with coronary heart disease (CHD) from the U.S. National Health and Nutrition Examination Survey (NHANES, 2003–2018) and evaluated the relationship between dietary inflammatory index (DII) and risk of all-cause mortality [90]. The mortality rate was significantly higher in patients with a high DII score. Further, women overall had higher DII scores compared to men. This suggests that managing dietary inflammation in CHD is imperative, especially in women [90]. Collectively, these findings indicate that high inflammatory diets contribute to CVD.

### 4.2. Anti-Inflammatory Diets

Given the role of inflammation in CVD, anti-inflammatory diets have emerged as promising strategies for CVD prevention. Specifically, diets rich in whole grains, green leafy vegetables (spinach and kale), nuts (almonds), and fruits (berries and apples), which are abundant in fiber, polyphenols, and antioxidant vitamins, are thought to improve CVD outcomes and reduce inflammation.

Anti-inflammatory diets known to improve CVD outcomes and reduce inflammation include the Dietary Approaches to Stop Hypertension (DASH) nutrition plan and Mediterranean diet (MD). The DASH nutrition plan recommends individuals consume whole grains, potassium-rich fruits, vegetables, lean meats, low-fat dairy products, and lower sodium intake [91]. The MD generally refers to the eating habits of people from countries bordering the Mediterranean Sea. The MD consists of consuming fruits, vegetables, whole grains, nuts and seeds, with extra virgin olive oil serving as the primary fat source and moderate amounts of dairy products, fish, eggs, and poultry [92].

In a DASH-Sodium randomized controlled trial in 2023, 412 adults with hypertension with no prior history of cardiometabolic disease were placed on a DASH diet (anti-inflammatory and low sodium) or a typical Western diet (pro-inflammatory) for 12 weeks [91]. Specific circulating biomarkers for cardiac injury and inflammation including high-sensitivity cardiac troponin I (hs-cTnI), *N*-terminal pro-B type natriuretic peptide (NT-proBNP), and high-sensitivity C-reactive protein (hs-CRP) were all measured. While NT-proBNP, a crucial biomarker for the diagnosis of heart failure [93], was unchanged between the two dietary groups, hs-cTnI and hs-CRP levels were significantly reduced in the DASH dietary cohort, supporting the cardioprotective and anti-inflammatory benefits of the DASH diet [91].

Consistent findings were reported in a systematic review and meta-analysis combining data from 18 randomized controlled trials involving 2602 participants, which revealed that anti-inflammatory diets lower the risk for CVD. Specifically, anti-inflammatory diets were associated with significantly reduced blood pressure, LDL cholesterol, and hs-CRP levels. In contrast, pro-inflammatory diets comprised of processed and ultra-processed foods increased systemic inflammation and CVD risk [94].

Recent human clinical studies have also supported the beneficial role of MD supplemented with nuts such as almonds in decreasing inflammation and being cardioprotective [95,96]. The PREDIMED trial, a multicenter randomized controlled trial testing MD for primary CVD prevention, involved 7477 adults with high CVD risk. Participants assigned to either an MD supplemented with mixed nuts (almonds, walnuts, hazelnuts) or an MD supplemented with extra-virgin olive oil experienced reduced cardiovascular events compared to those on the low-fat control diet [95]. Another randomized control trial conducted by Osorio-Conles et al., involving 38 obese women, found that adhering to a MD supplemented with almonds for 3 months decreased circulating inflammatory markers and LDL cholesterol, while increasing anti-inflammatory M2-like macrophage markers and lipolysis [96].

MD adherence has also been associated with lowering kidney stone risk. A 2020 longitudinal study involving 3 U.S. cohorts known as the Health Professionals Follow-Up Study, Nurses’ Health Study I, and the Nurses’ Health Study II, found that while urinary oxalate increased with higher MD adherence, protective factors such as urine volume and citrate also increased, and calcium excretion did not, contributing to a lower kidney stone incidence [97]. In another 2017 longitudinal study, 16,094 adults from Spain showed that higher MD adherence significantly reduced kidney stone risk. This study further confirmed the inverse association between dairy consumption and kidney stone risk [98].

Taken together, these findings suggest the DASH and MD as being cardioprotective, reno-protective, and anti-inflammatory. These diets primarily exert their cardioprotective effects by increasing HDL levels and reducing inflammation. Furthermore, anti-inflammatory diets, which include some oxalate rich foods such as nuts and green leafy vegetables, may include dairy products that provide dietary calcium, which decreases oxalate absorption in the gut and renal oxalate excretion. Additionally, the high fiber content of anti-inflammatory diets may promote hydration, which helps to flush metabolic toxins such as oxalate from the body. Overall, diets rich in whole, minimally processed foods reduce systemic inflammation and lower CVD risk.

### 4.3. Oxalate Diets

Oxalate is a uremic toxin and the conjugate base of oxalic acid that can be obtained from dietary sources or produced endogenously [99]. High concentrations of oxalate are present in plant-based foods such as spinach, beets, raspberries, potatoes, almonds, and peanuts [19,20]. Many of these foods are considered to be anti-inflammatory and cardioprotective. Endogenous oxalate synthesis occurs in the liver from precursors such as the amino acids, hydroxy-L-proline (HLP) and glycine, which are abundant in collagen-rich animal products [100].

Oxalate readily binds to cations such as sodium and calcium to form soluble or insoluble salts, respectively. Soluble oxalate is absorbed into the circulation [101,102], whereas insoluble calcium oxalate can crystallize in soft tissues and cause injury [103]. The amount of dietary calcium is essential, as oxalate absorption is related to calcium concentration [104]. Diets containing a high calcium load have reduced intestinal oxalate absorption because the insoluble CaOx molecules formed in gut are not absorbed [105]. Excess oxalate is primarily excreted by the kidneys [20,101,106,107] and impaired renal function or oxalate metabolism can lead to increased systemic oxalate levels, which can be toxic. Because calcium oxalate is the main constituent of most kidney stones, individuals at risk for nephrolithiasis are advised to limit oxalate-rich foods and increase calcium consumption. This extends to beverages high in fructose, such as soda which are linked to kidney stone formation, as fructose can increase calcium and oxalate excretion [108,109]. In addition, tap and bottled water high in minerals like calcium and magnesium may reduce kidney stone risk [110]. Foods high in oxalate can also have high levels of calcium [111], suggesting some oxalate-rich foods may pose more risk than others. Importantly, portion size, food combinations, and calcium intake can reduce oxalate absorption. This suggests that some oxalate rich foods such as nuts remain consistent with eliciting cardioprotective and anti-inflammatory effects.

Importantly, kidney stone disease is independently associated with increased CVD risk [21,27,112,113,114,115,116,117,118,119]. Kidney stones also cause kidney injury through recurrent urinary obstruction, infection and crystal-associated tubular damage, which triggers renal inflammation and fibrosis [120]. Thus, diets rich in oxalate could pose health risks for vulnerable populations, such as individuals with impaired renal function or a history of kidney stones. Additionally, some studies suggest calcium supplementation increases the risk of coronary heart disease [121], implying increased dietary calcium to combat oxalate load may be ill-advised in select populations. This presents a concern because many foods promoted as heart healthy and anti-inflammatory are also high in oxalate. This raises important questions about how oxalate-rich foods may exert context-dependent effects on immune activation and vascular health. We have reported that moderate to high levels of dietary oxalate induces ROS in monocytes and macrophages in healthy adults [22,23] and others have linked dietary oxalate to CVD risk in human cohorts and animal models [122,123]. Collectively, these observations highlight dietary oxalate as a potentially overlooked modifier of inflammation and redox status within otherwise cardioprotective dietary patterns, motivating more investigation into oxalate’s role in modulating CVD risk.

## 5. Oxalate, Inflammation, and Oxidative Stress

Oxalate has well established roles in inflammation [24,124,125,126], oxidative stress [127], and immune activation via the inflammasome, MAPK, complement, and NF-κB signaling pathways [128]. In recent years, growing attention has focused on the effects of oxalate on cells of the innate immune system, particularly monocytes and macrophages [22,23,129,130,131,132]. Notably, the majority of this work has centered on renal pathology, with limited investigation into the broader implications of oxalate-induced immune activation in the context of CVD.

In murine renal cell models, oxalate activates the NLRP3 inflammasome, triggering inflammatory responses that promote CaOx crystal formation and systemic inflammation [125]. CaOx crystals similarly activate the NLRP3 inflammasome in dendritic cells [133] and downstream inflammatory responses [125]. Oxalate can also induce endoplasmic reticulum stress, leading to NF-κB activation and increased ROS generation [134]. Systemic oxalate acts as a DAMP, resulting in tissue injury, oxidative stress, and inflammation [125,135]. In fact, impaired oxalate excretion results in widespread systemic inflammation and kidney injury in both rodents and human patients [24,125,126]. Further, CaOx monohydrate (COM) crystals activate MAPK and ROS/Akt/p38 signaling, leading to a disruption in tight junctions and further inflammation [136,137].

Beyond renal tissues, oxalate directly alters innate immune function. In human monocytes, oxalate polarizes human macrophages towards an M1 phenotype (pro-inflammatory) [138], while reducing M2 (anti-inflammatory) populations. M1 macrophages promote CaOx crystal deposition, whereas M2 macrophages are essential for removing and clearing CaOx crystals through phagocytosis [138,139,140,141,142,143]. Oxalate also increases mitochondrial ROS levels and impairs mitochondrial function in monocytes and macrophages [135]. Elevated ROS production is linked to CaOx crystal formation, growth, and retention [22,135]. In a dietary intervention study in healthy adults, we demonstrated that a dietary oxalate load alters monocyte and macrophage metabolism, redox status, and transcriptomics [22]. Intriguingly, IL-10 signaling was one of the most downregulated pathways in monocytes following oxalate consumption and is consistent with impaired resolution of inflammation. Our complementary studies conducted in Sprague Dawley rats showed that HLP, an oxalate precursor, induced renal inflammation and pro-inflammatory M1 macrophage populations [24,144]. Despite growing evidence of oxalate influencing innate immune activation, the impact of oxalate on the adaptive immune system remains poorly defined. Although sustained inflammation can recruit and activate T and B cells (adaptive immunity), oxalate-specific impacts have not been clearly delineated. Additional studies are needed to determine whether oxalate directly modulates adaptive immunity. Because chronic inflammation is a central driver of CVD, oxalate-mediated immune activation provides a biologically plausible link between dietary oxalate and CVD. Importantly, the immune-mediated vascular effects of oxalate remain theoretical based on the limited direct evidence currently available.

## 6. Oxalate and CVD

Since oxalate is the primary component of most kidney stones, the majority of oxalate-related studies have centered on renal pathology. However, a growing number of scientific reports suggest that oxalate-induced inflammation and oxidative stress activate cellular pathways that overlap with those central to CVD. Although direct evidence linking kidney stones or oxalate to CVDs remains limited, this understudied area of research highlights the importance of investigating potential shared pathological mechanisms. Here, we examine clinical and experimental data connecting oxalate to a number of CVDs and how oxalate may contribute to the cardio-renal axis. To improve clarity, the following sections distinguish between direct cardiovascular effects of oxalate, cardiovascular effects secondary to oxalate-induced kidney injury, and immune-mediated mechanisms that remain theoretical based on available evidence.

### 6.1. Atherosclerosis

The presence of CaOx crystals within atherosclerotic plaques was first reported in postmortem coronary specimens from the National Neurologic AIDS Bank, including cases without documented renal failure [145]. This suggested that vascular oxalate deposition could occur independent of kidney dysfunction and may exacerbate conditions within the atherosclerotic lesion. Consistent with this thought, oxalate can induce calcium influx in endothelial cells, which could initiate an atherogenic microenvironment, endothelial dysfunction, and promote plaque formation [25] (Figure 1). Disruption of oxalate homeostasis may further promote atherosclerosis, as demonstrated by a recent study using *Agxt* and *ApoE* double knockout mice. Alanine-glyoxylate aminotransferase (AGXT) plays a vital role in oxalate homeostasis by converting the waste product glyoxylate to glycine, preventing its buildup and conversion to oxalate [146,147]. Similarly, APOE contributes to plasma lipid regulation by mediating the transport of cholesterol- and triglyceride-rich lipoprotein particles to the liver for clearance [148,149]. Liu et al. determined that *ApoE* and *Agxt* double knockout male mice have increased plasma total cholesterol levels, atherosclerotic lesions, CCL5 expression (pro-inflammatory-chemoattractant cytokine) and oxidative stress [18]. In addition, these mice had a significant upregulation of pro-atherogenic inflammatory genes and cytokine signaling pathways compared to control *ApoE* knockouts [18].

### 6.2. Vascular Calcification

Vascular calcification (VC) is a common feature found in advanced atherosclerosis and chronic kidney disease (CKD) characterized by calcium phosphate crystal deposition in the vascular wall [150]. In a large cohort of kidney dialysis patients, approximately 90% of patients experienced VC and coronary artery calcification that was associated with an increased risk of CVD morbidity and mortality [151]. VC involves osteogenic differentiation, activation of JAK/STAT signaling, inflammatory cytokines, abnormal mineral homeostasis, and matrix remodeling [152,153,154,155,156]. In renal tubular epithelial cells, oxalate induces osteogenic differentiation and calcification via JAK2/STAT3 signaling pathway [157]. Further, oxalate contributes to VC by promoting CaOx crystal deposition in vascular tissue [145]. Elevated plasma oxalate levels also increase endothelial intracellular calcium concentrations and prevent re-endothelialization [25]. Clinically, Agatston scoring quantifies aortic calcifications, and higher Agatston scores have been shown to be significantly associated with reduced estimated glomerular filtration rate in non-dialysis patients with stable chest pain and individuals with CKD [158,159]. In a recent report, the Agatston score was used to compare aortic calcification and kidney stone size and showed that an increase in severity of aortic calcification correlated with an increase in kidney stone size [160]. While oxalate is documented to be in vascular tissues, direct mechanistic insights into how it alters vascular structures, especially in VC remains limited and warrants further study.

### 6.3. Coronary Artery Disease

CAD is driven by atherosclerotic narrowing of the coronary vessels and leads to chest pain (angina pectoris), abnormal heart rhythms, and MI (heart attack). CAD contributes significantly to morbidity and stands as a third leading cause of mortality worldwide [161,162]. Healthy nutrition and lifestyle changes can minimize CAD risk [163]. In a follow-up study of 4564 kidney stone formers and 10,860 matched controls, there was a 38% increased risk of MI for stone formers, which remained at 31% after adjusting for CKD and other comorbidities [164]. Additional studies also show an increased risk of MI, revascularization, and percutaneous transluminal coronary angioplasty/coronary artery bypass grafting in kidney stone formers [27,114,119,165]. Taken together, these findings highlight a consistent epidemiological association, yet the mechanistic basis through which oxalate may influence CAD risk remains unknown.

### 6.4. Stroke

Stroke is the second leading cause of death globally [166] and frequently results from atherosclerotic plaque rupture and thromboembolism (blood vessel obstruction through dislodged clots). Oxalate may contribute to stroke risk through its pro-inflammatory and pro-thrombotic effects [167]. Several population-based studies in Taiwan and Canada reported an increased risk of stroke among patients with urinary stones [27,168,169], although this association varies across patient subgroups. A Swedish study showed that individuals with normal blood pressure did not have a significant association between urinary stones and stroke risk [170]. A case report of a 27-year-old man with Primary Hyperoxaluria Type I revealed circulating microemboli, likely formed from excess serum oxalate that exceeded the calcium oxalate supersaturation threshold [171]. Brain imaging showed rapid mineralization in the stroke-affected brain areas, suggesting direct oxalate deposition in the damaged tissue. Oxalate-induced endothelial dysfunction [25,172] and impaired cerebral blood flow may further susceptibility to plaque rupture and ischemic events. Despite these emerging reports and supporting evidence, mechanistic insight into the relationship between oxalate and stroke remains limited.

### 6.5. Hypertension and Sudden Cardiac Death

Hypertension, also known as high blood pressure, is a condition that arises due to elevated blood pressure in the blood vessels. It is also a major risk factor for CVD and is linked to oxalate metabolism. Urinary oxalate levels were reported to be elevated in individuals with hypertension compared to normotensive controls [173]. Further, an 8-year prospective study established an association between high dietary oxalate and low calcium intake with an increased risk of developing hypertension [174]. SCD occurs when there is an abrupt halt in the function of the heart and is considered a cardiac arrhythmia condition. It affects approximately 1000 people each day in US with a survival rate of just 10% [175]. In a post hoc analysis of patients on hemodialysis, increased serum oxalate levels were correlated with an increased incidence of SCD [26]. Together, these studies suggest that a diet rich in oxalate and low in calcium may contribute to vascular and arrhythmic risk in vulnerable populations. Additional studies are warranted to further define mechanistic pathways influenced by high oxalate intake.

### 6.6. Renal Pathways Linking Oxalate to CVDs

The observed association between elevated serum oxalate and sudden cardiac death in dialysis patients [26] highlights a broader cardio-renal framework in which the kidney governs systemic oxalate exposure and, in turn, shapes CVD risks. The majority of endogenous and dietary oxalate is eliminated through glomerular filtration and tubular secretion [99], with the proximal tubule serving as a major site of active oxalate handling. As renal function declines, oxalate shifts from a dietary or metabolic byproduct to a retained systemic toxin. Consistent with this concept, reduced renal function is associated with oxalate accumulation in the blood and crystal deposition in soft tissues [176] including the kidneys, which promotes oxidative stress and inflammation.

This oxalate retention state is most pronounced in advanced CKD and dialysis, where kidney function is severely impaired. Notably, dialysis patients who retain urine output have lower plasma oxalate levels [177], underscoring the protective role of residual kidney function. CKD patients with elevated serum oxalate exhibit increased cardiovascular mortality [27,115,178]. These associations are particularly relevant in late-stage kidney disease, where VC and coronary artery calcification are highly prevalent and linked to increased CVD morbidity and mortality [151]. Supporting biologic plausibility for direct vascular effect in uremic states, uremic atherosclerosis models demonstrate that elevated plasma oxalate exacerbates vascular inflammation and oxidative injury [127].

Oxalate-associated cardiovascular risk is not confined to overt renal failure. CaOx crystals were identified within atherosclerotic plaques in postmortem coronary specimens [145], including cases without documented renal failure. These findings suggest that vascular oxalate deposition can occur independent of reduced glomerular filtration. Additionally, increased MI and stroke risk in kidney stone formers [165] are partly independent of CKD in some analysis [164], further supporting the possibility that oxalate-associated processes influence vascular risk beyond reduced kidney function alone. Collectively, these findings support a cardio-renal model in which declining renal oxalate clearance increases systemic and vascular oxalate burden, amplifying oxidative and inflammatory injury and thereby accelerating cardiovascular risk from nephrolithiasis to advanced CKD and dialysis.

## 7. Future Directions

Overall, this narrative review highlights how oxalate may promote oxidative stress, inflammation, endothelial dysfunction, and VC in CVD. To date, oxalate research has focused primarily on renal disease and has identified many established pathways known to drive CVD. Yet, the role of dietary oxalate on cardiovascular outcomes remains undefined and warrants further investigation. As we move into an era of personalized medicine, perhaps we need to consider personalized dietary interventions.

Several clinical questions arise from the findings presented in this review. Given the known health benefits of some oxalate-rich foods, these questions need to be addressed before altering dietary recommendations to CVD patients. First, is high dietary oxalate intake harmful to individuals with existing CVD or those at elevated risk? Second, what amount of dietary oxalate is appropriate in a CVD patient’s diet? Third, are individuals that consume anti-inflammatory diets that emphasize oxalate-rich foods at greater risk? Fourth, do the other cardioprotective components of select oxalate rich foods (such as polyphenols) outweigh damaging effects of oxalate? If so, only a subset of oxalate-rich food may be detrimental to the at-risk populations. Finally, will low-oxalate diets alone or in combination with adequate dietary calcium intake (important for modulating oxalate absorption) benefit patients in select subgroups? Consideration should also be given to how oxalate-containing foods are consumed, including pairing them with protective components such as calcium-rich foods, as well as using appropriate cooking methods that can reduce oxalate content or limit its harmful effects. Lastly, given evidence that oxalate suppresses anti-inflammatory signaling and enhances oxidative stress in innate immune cells, does augmenting systemic anti-inflammatory responses mitigate oxalate-associated vascular injury?

Clinical trials targeting inflammation provide proof of concept that inflammatory pathways are modifiable drivers of cardiovascular risk. The Canakinumab Anti-inflammatory Thrombosis Outcome Study determined that inhibiting IL-1β (a pro-inflammatory cytokine) with canakinumab (monoclonal human anti-IL-1β antibody) reduced the recurrence of cardiovascular events in patients with a history of MI [179]. Notably, IL-1β is a downstream product of inflammasome activation, a pathway strongly implicated in oxalate-induced immune responses described above. Other anti-inflammatory therapies, such as colchicine and methotrexate, are also being investigated for their potential benefits in CVD [180]. Future studies should investigate the impact of controlled dietary interventions on inflammatory biomarkers and cardiovascular outcomes. In addition, mechanistic studies describing how oxalate affects vascular and immune cells are needed.

## 8. Conclusions

The intersection of oxalate biology with atherosclerosis, VC, and cardio-renal disease highlights the need to examine dietary oxalate within broader frameworks of cardiovascular risk and inflammation. Elucidating additional oxalate-mediated mechanisms in immune cells and the vasculature could inform anti-inflammatory strategies for CVD prevention and treatment or may guide dietary recommendations for vulnerable populations.

## Data Availability

The original contributions presented in the study are included in the article, further inquiries can be directed to the corresponding author.

## Abbreviations

The following abbreviations are used in this manuscript:

AGXT	Alanine-glyoxylate aminotransferase
Ca^2+^	Calcium
COM	calcium oxalate monohydrate
CVD	cardiovascular disease
CVDs	cardiovascular diseases
CKD	chronic kidney disease
CAD	coronary artery disease
CHD	coronary heart disease
DAMPS	damage-associated molecular patterns
DII	dietary inflammatory index
EDIP	empirical dietary inflammatory pattern
HSP70	heat shock protein 70
HMGB1	high-mobility group box 1
hs-cTnI	high-sensitivity cardiac troponin I
hs-CRP	high-sensitivity C-reactive protein
HLP	hydroxy-L-proline
IFN-γ	interferon-gamma
IL-1β	interleukin-1β
IL-8	interleukin-8
IL-16	interleukin-16
IL-18	interleukin-18
LDL	low-density lipoprotein
MMPs	matrix metalloproteinases
MD	Mediterranean diet
MAPK	mitogen-activated protein kinase
MCP-1	monocyte chemoattractant protein-1
MI	myocardial infarction
NETs	neutrophil extracellular traps
NLRP3	NLR family pyrin domain containing 3
NT-proBNP	*N*-terminal pro-B type natriuretic peptide
NF-κB	nuclear factor-kappa B
oxLDL	oxidized LDL
PAI-1	plasminogen activator inhibitor-1
ROS	reactive oxygen species
S100A12	S100 calcium-binding protein A12
SCD	sudden cardiac death
TNF-α	tumor necrosis factor-alpha
VC	vascular calcification
